# Melanoma predisposition—A limited role for germline *BRCA1* and *BRCA2* variants

**DOI:** 10.1111/pcmr.12833

**Published:** 2019-10-28

**Authors:** David J. Adams, David Timothy Bishop, Carla Daniela Robles‐Espinoza

For several decades, the role of germline *BRCA1*/*BRCA2* variants in predisposition to melanoma has been controversial. Several groups have reported a positive association between *BRCA* variant status and melanoma formation, while other studies have failed to find an association (Gumaste et al., 2015). In the same way, analyses of familial melanoma cohorts have reported germline pathogenic alleles of *BRCA2* (Gumaste et al., 2015; Tuominen et al., 2016) providing some evidence that germline variants in these genes might contribute to disease pathogenesis—but do they?

The potential involvement of germline *BRCA* variants in melanoma susceptibility has profound implications for patient management and screening, since there are now agents such as poly‐(ADP‐ribose)‐polymerase (PARP) inhibitors (Farmer et al., 2005) that could be used to treat tumours from carrier patients, or even potentially prophylactically to prevent tumour formation. Further, several commercial vendors promote *BRCA1/2* testing in patients with a history of melanoma, so it is important for patients and the healthcare system to establish whether such testing is warranted, not only because of the associated cost but also because genetic testing can be associated with significant patient anxiety.

The tumour spectrum associated with germline *BRCA* variants has largely been elucidated by examining the cancer history of carriers and presumed carriers in families identified because of multiple cases of breast or ovarian cancer. Of course, other cancers occur in these families both by chance but also potentially because the germline variant increases cancer risk. Typically, the observed numbers of cancers in carriers are compared to the incidence of these cancers in the general population, but the power of individual studies is usually low because the number of actual carriers is limited. So why might the association between *BRCA* alleles and melanoma be inconsistent? Firstly, it is possible that *BRCA* alleles have no effect and that chance variation provides a statistical signal suggesting an association between these variants and melanoma. Secondly, that there is a modest effect and again chance variation plays a role. Thirdly, that any effect is limited to a subset of *BRCA* variants (e.g., the Ovarian Cancer Cluster Region which is associated with a much higher relative risk of ovarian cancer than other variants in *BRCA1* or *BRCA2* (Rebbeck et al., 2015)) and that the variant profile differs in different cohorts. Finally, melanoma risk is not uniform across populations because common melanoma risk factors including pigmentation and nevi (which are unrelated to *BRCA* genotype) are polygenic. These variants, such as the *R* alleles of *MC1R*, are known to be able to influence the risk of melanoma development even in individuals with established high‐penetrance melanoma predisposition alleles such as pathogenic variants in *CDKN2A*. Identifying a suitable control population of a sufficient size, that is well‐matched to carriers and has the same melanoma polygenic risk profile, poses an additional problem and can confound the analysis.

In a large‐scale and exhaustive study of cancers by the IMPACT team at Memorial Sloan Kettering Cancer Centre by Jonsson and collaborators, where cancer patients being treated at the hospital were uniformly ascertained and whose tumour and germline genomic sequences were systematically analysed by targeted sequencing, the authors were able to show that if germline *BRCA1* or *BRCA2* variants do contribute to the population burden of melanoma predisposition then this contribution is modest. What is particularly special about this study is that the scale of the patient cohort (17,152 patients, 55 tumour types, 621 melanoma patients) meant that statistically robust conclusions could be derived. By analysis of these patients, clear and unequivocal associations between germline and somatic *BRCA1/2* variants and ovarian, prostate, breast and pancreas cancer (the BRCA‐associated cancer types) were observed, as well as a potentially novel association between somatic mutations in *BRCA2* and uterine sarcomas. Indeed, it was only in the BRCA‐associated cancer types that genomic analysis was able to find a significant level of somatic loss‐of‐heterozygosity (LOH) consistent with the recessive model of tumour suppressor gene function. In comparison, when the same analysis was done on non‐BRCA‐associated cancer types (which includes melanoma), tumour‐specific loss of the germline pathogenic allele instead of the wild‐type allele was observed more frequently, further suggesting that loss of *BRCA1* or *BRCA2* did not contribute to these cancers. Moreover, it was only in ovarian, prostate, breast and pancreas cancer that the diagnostic single nucleotide pattern associated with loss of homologous recombination could be observed, with only a modest increase of this signature in non‐BRCA‐associated cancer types with biallelic *BRCA* inactivation. For the other cancer sites, only between 0.5% and a few per cent of cases had a germline *BRCA* variant (1% for melanoma). These figures are broadly in keeping with the make‐up of the population studied of whom more than 18% were of Ashkenazi descent, a population known to have a high carrier rate of *BRCA* variants. As for the 621 melanoma patients in the study, there were 258 females and 363 males, and the mean and median age at first diagnosis were 56.9 and 59 years old, respectively, with age unknown for 26 patients.

Another key observation of this study was that while patients from BRCA‐associated cancer types that had a germline, potentially pathogenic BRCA variant derived greater clinical benefit from PARP inhibitor treatment than those without these variants, this was not true for patients with non‐BRCA‐associated cancers. Although their analysis only included one melanoma patient, it suggests that germline testing should not be the only criterium by which patient treatment is chosen and that other aspects such as tumour lineage might play an equal, if not more important, role in therapy response.

Are there any limitations of the current study? In the context of melanoma, the authors grouped together all melanoma subtypes including cutaneous, uveal, acral and mucosal melanoma. It is known that the genomes of these different melanoma subtypes are distinctly different, with the genomes of uveal, acral and mucosal melanoma being characterized by a high level of copy number gains and losses, while the genomes of cutaneous melanoma are replete with UV‐induced C > T mutations. By “pooling” all subtypes together in this way it is possible that any statistical signal that might link *BRCA1* or *BRCA2* variants to rarer melanoma subtypes could be diluted. That said, on balance, it seems unlikely that germline *BRCA1* or *BRCA2* variants profoundly contribute to any melanoma subtype. It should be noted that large‐scale studies of this type cannot conclude that *BRCA1* or *BRCA2* variants never contribute to melanoma formation, just that at a population level they are not major contributors to the burden of disease.

In summary, Jonsson et al. have made a significant contribution to the debate on the involvement of *BRCA1* and *BRCA2* variants to melanoma predisposition and provide a reminder that we must constantly revisit gene‐disease associations so as to better counsel patients about their care.

## References

[pcmr12833-bib-0001] Farmer, H. , McCabe, N. , Lord, C. J. , Tutt, A. N. J. , Johnson, D. A. , Richardson, T. B. , … Ashworth, A. (2005). Targeting the DNA repair defect in BRCA mutant cells as a therapeutic strategy. Nature, 434, 917–921. 10.1038/nature03445 15829967

[pcmr12833-bib-0002] Gumaste, P. V. , Penn, L. A. , Cymerman, R. M. , Kirchhoff, T. , Polsky, D. , & Mclellan, B. (2015). Skin cancer risk in BRCA1/2 mutation carriers. British Journal of Dermatology, 172, 1498–1506. 25524463 10.1111/bjd.13626 PMC5785081

[pcmr12833-bib-0003] Rebbeck, T. R. , Mitra, N. , Wan, F. , Sinilnikova, O. M. , Healey, S. , Mcguffog, L. , … Andrulis, I. (2015). Association of type and location of BRCA1 and BRCA2 mutations with risk of breast and ovarian cancer. JAMA, 313, 1347–1361. 25849179 10.1001/jama.2014.5985 PMC4537700

[pcmr12833-bib-0004] Tuominen, R. , Engstrom, P. G. , Helgadottir, H. , Eriksson, H. , Unneberg, P. , Kjellqvist, S. , … Höiom, V. (2016). The role of germline alterations in the DNA damage response genes BRIP1 and BRCA2 in melanoma susceptibility. Genes, Chromosomes & Cancer, 55, 601–611. 27074266 10.1002/gcc.22363

